# Mechanical Dentistry

**Published:** 1880-12

**Authors:** E. M. Flagg

**Affiliations:** New York.


### ARTICLE III.

### *Mechanical Dentistry.*

BY E. M. FLAGG, D. D. S., NEW YORK.

(Read before the Connecticut Valley Dental Society.)

It has become to-day a very important question for our profession to decide, whether celluloid is to be a failure or a success as a dental plate. Of late there has existed a strong disposition on the part of many members of the profession to evade the subject of artificial dentistry, or ignore it entirely. Its discussion has been more or less excluded from dental associations, and in some cases motions have been made to deny it a position altogether as a branch of dentistry. One might almost suppose, to hear certain gentlemen speak of artificial dentistry in the manner they do, that they had so far outgrown it, and it had become a matter so simple and all its requirements had been so completely fufillled, that mental energy bestowed in that direction was a mere waste of brain-power. I claim the contrary. Its requirements have not been fulfilled. An artificial denture is supposed to represent the natural organ in a manner that its expression, character and effect will harmonize with the individual for whom it is made. In other words, that it will be an exact portrait of what the patient's teeth would have been had he preserved them in health to the time when the artificial substitute was inserted. There are many gentlemen in the profession who are wearing artificial dentures, and judged by the test just named there is not one denture in ten but that could be picked to pieces by perfectly legitimate criticism. So much for the requirements of artificial dentistry having been fulfilled. Remarks in this direction might be continued indefinitely.

The necessity for a higher standard of artistic culture in this direction cannot be ignored. It is useless to prate about the number of teeth that are being saved annually.

That does not help those who have lost their teeth one particle, and we all know it is no uncommon remark on the part of patients, that it is impossible to make a set of artificial teeth like natural. So far as rubber is concerned this remark is true. You cannot get individuality from a row of moulded porcelain blocks. As well might you expect an artist to make a portrait by selecting from a quantity of manufactured noses, eyes, ears, etc., and joining them together on a canvass. To obtain individuality in work the artist must not be restricted in his ability to arrange any portion of the material which he uses in any manner that he may desire. He may take a single plain tooth and carve and polish it in any form he desires, provided the material be sufficient; but a moulded block, as it comes from the manufacturer, cannot be made by any amount of artistic manipulation to alter its rigid, lifeless expression. So far is this assertion true, that I have had agents of dental depots tell me that they could always detect whether the wares exhibited in the mouths of those they met while traveling were from the moulds manufactured by the house they represented or not. What a comment to be made in a country where our profession is supposed by some to have reached the highest plane of development!

Celluloid has not this defect named in connection with rubber. Single, plain teeth can be used, and the artist is not restricted in giving the highest expression and character to his work. If we appreciate the requirements of his case there is nothing to prevent him from fulfilling them if he uses celluloid; so I say again, that there is no question so important for the profession to decide as whether celluloid is to be a success or a failure. The defects found to accompany the use of celluloid are as follows: (1) A wrapping of the material either in the process of moulding the plate or soon after the plate is inserted. (2) Discoloration of the material around the necks of the teeth, soon to be followed by discoloration of the entire plate and subsequent softening of the plate. (3) After sufficient softening the plate itself often breaks, which breakage is assisted by the disposition of the material to warp, and the teeth are inclined to drop off by the celluloid shrinking from the pins and the softening of the material around the pins.

The plate at first introduced itself upon the notice of the patient by a constant taste of camphor in the mouth, and to some patients this effect is very dispiriting, but as the plate becomes discolored and softened, the taste and smell of the celluloid is disgusting in the extreme, and the patient is inclined to condemn a material whose sole defect may lie in the manner in which it is worked. If we go on working it in the manner which we have been taught, celluloid is to be a failure, because there has been no apparatus that will fulfill the conditions required to the production of a perfect plate.

These conditions are as follows: (1) No steam, oil, or other foreign substance must be allowed to come in contact with the material while it is plastic, or it will discolor. (2) During the process of pressing the celluloid one portion of the blank must not be one degree colder than another portion, or it will warp. (3) The material must have its form changed at a heat much higher than is now used, otherwise it will still have a tendency to return to its original position, or, in other words, warp. This superior heat must be obtained in an apparatus that is airtight, for if the superheated material can obtain oxygen either in the form of vapor or a current of air, it will surely burn.

Let us now briefly consider the various apparatus that have been used for working celluloid and we shall see how unfit they are for producing a perfect plate, or if capable of producing good results, we shall see how the labor required will render them impracticable to the mass of the profession.

First, we have the glycerine machine, that produces a plate in which the celluloid had the benefit of a good soaking in this greasy, penetrating compound while the celluloid was in a softened condition, and the pins of the teeth had the additional advantage of being well greased, which left them in a condition admirably adapted to facilitate their future exit from the plate.

In the second place, we had the steam machine, with no thermometer and a valve in the place of it. Our experience with this instrument was that it produced a plate whose texture was not in any way improved by a volume of overheated steam rushing through it while in a plastic condition, and the contact between tooth and plate was anything but perfect. The plaster cast was often softened to such a degree by the hot steam and water that the fit of the plate would be destroyed. The investment would also soften to such an extent that the articulation would be badly impaired. The plunger, running in a steam packed cylinder, always left us doubtful as to whether the plate or the rubber of the cylinder was making resistance to our pressure, while such a thing as getting a fine dense texture to our celluloid was entirely out of the question. In fact, to enumerate all the defects of this instrument (so much vaunted by the Celluloid Company) would require more space than can be allowed to this entire article.

Another machine had a limited sale and was known as the Heintzmann dry oven. It purported to press celluloid at a dry heat, and was furnished with a large door, the opening of which served the double purpose of allowing the flask to be seen and the cold air at the same time to enter the flask. It also enables the operator to get one part of his flask very hot while the other remained comparatively cold, and it was with the greatest ease that he could burn one corner of his plate while the other remained so cold that the material would hardly flow. These bad results would be in part due to the misguiding influence of a thermometer that was placed on the top of the machine, which could only register the heat of the iron walls of the heater, and as the burner heated the iron walls and the flask was the last thing to receive heat, the thermometer was therefore of little use.

The fourth machine is known as the "Best," and although far better results could be obtained with this instrument than any of the others we have mentioned, there is probably no other machine that produced so many failures. "Dry, moist air" was not so bad as superheated steam for the texture of a celluloid plate; but any moisture is bad coming in contact with celluloid in a plastic state. It prevents elimination of whatever is volatile in the material, so that the material does not attain a good degree of hardness and cuts under the instrument in a manner known as cheesy. Perfect dryness of the flask is eminently desirable, and this cannot be satisfactorily obtained where there is any unevenness in the application of heat to the flask. Unless the heat is perfectly uniform the plaster is liable to powder and to crack, and becomes unfit for an investment.

In the Best machine, by constant watching and shifting the upper for the lower half of the flask continually, so that they would be alternately presented to the bottom of the oven, we might obtain a comparatively even heat. The directions said, "Wet the finger and touch the heated flask, as a laundress does a flat-iron, in order to see to what extent it would 'fiz.' " This primitive operation was supposed to serve the place of a thermometer and indicate the heat of the flask, but I have to record it as my experience that it was a very unreliable "thermometer," and I doubt that if all the evidence could be collected upon the subject of "finger fizzing" (or flask fizzing), it would lead to results sufficiently certain to warrant its use in the working of a substance so sensitive as celluloid is.

So much for the apparatus heretofore used, and if celluloid were dependent for its success upon the machines mentioned we should not have long to wait before it would be generally given up as a failure. A few men of the profession who could give their labor to celluloid as a specialty would have more or less success, but the mass of the pro fession in the smaller cities, who have to divide up their time between both branches of our specialty, would uot and could not afford to give their attention to a material whose best results were attended with uncertainty. For the most part they do not get large fees for work, and failure is with them a very serious consideration. They cannot afford to calculate upon making over plates again. The manufacturers cannot afford to bestow the same expense in the perfection of the "blanks" if their sale is to be narrowed down to a few specialists. Celluloid, as a material, has every advantage that can be hoped for. "Rubber" is known to be unfit for contact with the mucous membrane of the mouth, and "rubber poisoning" is familiar to nearly every dentist. Those who use rubber are victims to a restrictive monopoly. And yet there are probably fifty rubber plates made to one celluloid, and this proportion in favor of rubber will be greatly increased when the license tax upon it is removed.

I have said that celluloid, as a material, is the best that can be used in the mouth, and we will now describe an experiment to prove the truth of the assertion. Take a celluloid blank from the dental depot, say number six and a half full upper (this number covers as much surface as any,) pour plaster inside of it, making a model for the blank to rest upon. The blank will fit the plaster model, with the exception of the slight difference to be expected from the "setting" of the plaster. Now, if we keep the blank and the plaster model for six months or a year there will be no change in the fit. The Celluloid Company have not boiled the blank in grease or glycerine. They have not subjected it to a current of superheated steam, nor have they cooked it in an oven that would heat one part of it more than another. We may take the same blank and put it in soak in the promiscuous contents of our saliva-pump, and there it may soak for six months at a temperature of 100°F. with no change to either color or form. We will now take another blank and mould it in the steam heater upon a plaster cast. We will not put any teeth on it. As our plaster cast will be probably destroyed in the process of pressing, we shall have to make another one to preserve for the purpose of our experiment. The first effect we note is that our steam-moulded plate will smell strongly of camphor and cut under the instrument in somewhat the manner of hard cheese. In about three days the smell of camphor will have left the piece, and while we congratulate ourselves on that effect there will be another effect upon which we cannot congratulate ourselves, and that is that the steam moulded piece of celluloid will not fit the cast at all. So much for the effect of steam upon the fit. If we now take our steam-moulded piece of celluloid and put it in soak in the contents of the saliva-pump, we shall find that in one week it will begin to discolor, and in a month will present an appearance as disagreeable as bow-spring rubber.—*Dental Miscellany.*

